# The Culinary Acceptance of Edible Insects: The Mediating Roles of Food Neophobia and Food Interest in the Context of Perceived Future Food Insecurity

**DOI:** 10.3390/insects17070748

**Published:** 2026-07-22

**Authors:** Gökhan Onat, Yusuf Karakuş, Büşra Elem Yıldrım

**Affiliations:** 1 Department of Gastronomy and Culinary Arts, Ardeşen Tourism Faculty, Recep Tayyip Erdoğan University, Rize 53400, Türkiye; gokhan.onat@erdogan.edu.tr; 2 Department of Tourism Management, Ardeşen Tourism Faculty, Recep Tayyip Erdoğan University, Rize 53400, Türkiye; 3 Department of Gastronomy and Culinary Arts, Institute of Graduate Studies, Recep Tayyip Erdoğan University, Rize 53400, Türkiye

**Keywords:** edible insects, entomophagy, insect-based foods, culinary acceptance, recipe incorporation intention, food neophobia, food interest, perceived future food insecurity, sustainable protein, gastronomy students, Protection Motivation Theory

## Abstract

Edible insects are a promising source for sustainable protein transformation due to their low environmental impact, high nutrient density, and suitability for circular food systems; however, despite their high technical potential, culinary and societal acceptance remains low. While the literature has largely focused on consumer acceptance, culinary professionals’ intention to incorporate edible insects into recipes has been understudied. This study examines the direct and indirect associations of perceived future food insecurity with the intention to incorporate edible insects into recipes, mediated by food neophobia and food interest. Protection Motivation Theory was used as a guiding theoretical lens to conceptualize perceived future food insecurity as a threat-related contextual perception, rather than as a full test of the theory. Data were collected from 400 culinary students across seven regions of Turkey; confirmatory factor analysis and structural equation modeling were applied. The direct effect of perceived future food insecurity on recipe incorporation intention was not significant (β = −0.015). However, perceived future food insecurity was negatively associated with food neophobia (β = −0.212) and positively associated with food interest (β = 0.138); food neophobia was negatively associated with recipe incorporation intention (β = −0.225), whereas food interest was positively associated with it (β = 0.715). Both indirect effects were statistically significant, indicating indirect-only mediation. The findings provide context-specific evidence extending the edible-insect literature from personal consumption intention toward professional recipe incorporation intention.

## Simple Summary

The contribution of edible insects to sustainable food systems is made possible not only by their production and environmental benefits, but also by culinary professionals incorporating these ingredients into recipes and menus. This study focuses on culinary students, a critical intermediary group in the culinary adoption of insects, and examines how their perceptions of future food insecurity are associated with their intentions to incorporate edible insects into recipes. Data were collected via an online survey from 400 students across seven regions of Turkey and analyzed using structural equation modeling. The findings indicate that perceived future food insecurity is not directly related to the intention to incorporate edible insects into recipes; rather, it is indirectly linked through lower food neophobia and higher food interest. The results highlight the potential importance of culinary education; previous research further suggests that low-visibility processed forms may facilitate acceptance.

## 1. Introduction

Global food systems are under increasing pressure due to population growth, climate change, limited natural resources, and changing consumption patterns. According to United Nations projections, the world’s population is expected to exceed nine billion by the middle of the century; this will lead to a significant increase in protein demand in particular [1]. Since traditional livestock farming is a production method characterized by high levels of greenhouse gas emissions and intensive water and land use, questions have been raised about whether the current production model can sustainably meet future demand [2,3]. In this context, academic and industry interest in alternative protein sources that can provide higher nutritional value with fewer resources is growing rapidly [4,5].

Edible insects are a leading option among these alternative protein sources. Insects are rich in protein, essential amino acids, healthy fatty acids, and micronutrients; they can be produced with much lower feed conversion ratios and require less land and water compared to traditional livestock farming [1,6]. These characteristics make insects a technically robust solution not only for food but also for feed security. However, there is a significant gap between the technical sustainability of insects and their social acceptance. In Western and similar contexts without a tradition of entomophagy, acceptance levels of edible insects, and particularly the intention to incorporate them into food preparation, remain low [4,7].

The technical potential of edible insects is realized at the level of species, product form, and method of use. Species such as the mealworm (*Tenebrio molitor*), house cricket (*Acheta domesticus*), migratory locust (*Locusta migratoria*), buffalo weevil (*Alphitobius diaperinus*), and black soldier fly larvae (*Hermetia illucens*) are the most extensively studied taxa for both food and feed purposes. These species can be used whole, roasted, or in the form of ground flour or protein powder; they can also be incorporated into protein bars, bread and baked goods, pasta, snacks, and meat-like products. Nutritionally, insects are rich in high-quality protein, essential amino acids, polyunsaturated fatty acids, iron, zinc, and B vitamins; they also contain chitin, which functions as dietary fiber [6]. From a food safety perspective, key issues include allergenicity (particularly cross-reactivity with shellfish and mites), microbial load, processing and storage conditions, as well as regulatory compliance and consumer confidence [8,9]. In terms of sustainability, insects possess characteristics that make them a component of circular food systems through low feed conversion ratios, minimal water and land use, low greenhouse gas emissions, and the utilization of organic byproducts [1,2].

Edible insects may provide nutritional and environmental advantages, but their contribution to future food systems depends on social, sensory, and culinary acceptance. The technical potential of edible insects can be realized in food systems only if culinary professionals are willing to translate insect-derived ingredients into acceptable recipes, menu items, and products. As the chefs and food-and-beverage professionals of the future, gastronomy students represent a critical stakeholder group in this transition.

The factors limiting the acceptance of edible insects are largely psychological, cultural, and sensory in nature. Disgust, unfamiliarity, negative expectations regarding texture and appearance, concerns about hygiene and safety, and perceptions of cultural appropriateness are among the main barriers to the acceptance of insects as food [10,11,12]. One of the key concepts underlying these barriers is food neophobia, that is, a reluctance to try unfamiliar foods [13]. The way the product is presented also affects acceptance; using insects not in their whole form but in processed forms that are not visible, such as flour or protein powder, can reduce resistance [14]. Nevertheless, information and technical expertise alone may not be sufficient to eliminate neophobic reactions. By contrast, food interest, an individual’s general curiosity about food, cooking, and new culinary experiences, may function as a facilitating psychological resource that encourages engagement with unfamiliar ingredients such as edible insects; food neophobia and food interest are therefore treated in this study as distinct, though potentially coexisting, avoidance- and engagement-related psychological correlates.

In this regard, gastronomy students constitute a research group that is both specialized and theoretically significant. Gastronomy and culinary arts students will work in the future as chefs, menu developers, culinary educators, entrepreneurs, or food and beverage managers; consequently, they will serve as critical gatekeepers in bringing innovative food products to consumers [15,16]. Studies examining student chefs’ attitudes toward insect ingredients indicate that this group is open to innovative practices; however, the appearance, smell, and texture of insects negatively affect acceptance, while processed forms increase acceptance [17].

However, the literature on the acceptance of edible insects has largely focused on consumer acceptance, willingness to taste, purchase intent, and reactions of disgust or neophobia. Moreover, for insects to enter the actual food system, it is not enough for consumers to simply be willing to eat them; the product must be transformed into a recipe, a menu item, and a presentable format by chefs, culinary students, and food and beverage professionals. Therefore, recipe incorporation intention is a distinct behavioral outcome separate from consumer acceptance; it encompasses not only the intention to eat a food but also the intention to transform it into a product through culinary creativity, technical curiosity, and professional openness. This conceptual distinction has the potential to introduce the dimensions of foodservice implementation, culinary translation, and menu integration into the literature on edible insects. Taken together, three gaps remain: most research has focused on general consumers rather than culinary professionals; existing studies emphasize consumption or purchase intention rather than the intention to incorporate insects into recipes; and the indirect psychological mechanisms linking perceived future food insecurity to recipe incorporation intention remain largely unexamined.

At the theoretical level, this study uses the Protection Motivation Theory (PMT) not as a comprehensive theory test, but as an explanatory lens. PMT provides a psychological framework that shapes the processes by which individuals assess and cope with a threat when confronted with it [18,19]. Future food insecurity can be conceptualized as a forward-looking perception of threat related to the fragility of food systems. However, the PMT does not suggest that the threat alone automatically generates adaptive behavior. For the perception of a threat to become behaviorally meaningful, it must interact with coping evaluations and psychological readiness. This logic supports the central argument that perceived future food insecurity may be indirectly associated with the intention to incorporate edible insects into recipes through food neophobia and food interest. Since the model does not directly measure the coping components of PMT (response efficacy, self-efficacy, and response cost), the study is more accurately characterized as PMT-informed rather than PMT-based.

Recipe incorporation intention also differs from personal consumption intention in both actor role and behavioral content. Consumption intention concerns whether an individual is willing to eat an insect-based product, whereas recipe incorporation intention concerns whether a future culinary professional is willing to prepare, cook, serve, modify, or integrate insect-derived ingredients into recipes and menus. It therefore reflects professional implementation intention rather than personal dietary acceptance; although both constructs belong to the broader domain of behavioral intention, they are not interchangeable, and the adapted outcome measure used in this study was intended to capture professional culinary implementation rather than personal consumption (see Section 3.3).

In this context, the aim of this study is to examine the direct and indirect associations of perceived future food insecurity with gastronomy students’ intention to incorporate edible insects into recipes, through the mediating variables of food neophobia and food interest. This study contributes to the literature in several ways. First, it examines gastronomy students, an understudied group, rather than general consumer samples. Second, it focuses on recipe incorporation intention, a professional implementation construct, which is conceptually distinct from personal consumption intention (see Section 3.3), rather than personal consumption intention. Third, it positions perceived future food insecurity as a contextual threat variable in the adoption of insect-based cuisine. Fourth, it examines whether perceived future food insecurity is indirectly associated with recipe incorporation intention through food neophobia and food interest, using PMT only as a limited conceptual lens rather than as a fully tested theoretical model. The study’s contribution should be understood as primarily contextual rather than theory-building, given the cross-sectional design, the modest predictive power of perceived future food insecurity, and the substantial scale modification described below.

## 2. Theoretical Framework and Hypothesis Development

### 2.1. Edible Insects, Food Insecurity and Culinary Integration

Food systems are complex networks that span from agricultural production to consumption, encompassing the dimensions of production, distribution, access, and consumption. In recent years, these systems have been discussed not only in terms of supply capacity but also in terms of increasing uncertainties, access issues, and perceptions of vulnerability. For conceptual clarity, it is important to distinguish between the concepts of food security, food insecurity, food safety, food crisis, and food system vulnerability. Food security refers to the continuous physical, social, and economic access of all individuals to sufficient, safe, and nutritious food to lead an active and healthy life. Food insecurity, on the other hand, refers to the lack of such access; in other words, it describes the situation where individuals cannot consistently obtain sufficient, safe, nutritious, and culturally acceptable food. Food safety refers to food being hygienic and free from health risks; a food crisis refers to an acute and widespread disruption in access; and food system vulnerability refers to the system’s lack of resilience against economic, environmental, or social shocks. The concept focused on in this study is perceived future food insecurity, which reflects individuals’ perceived vulnerability regarding future access to food, dietary adequacy, food diversity, and food availability.

At this point, the perception of food insecurity can be considered a significant psychological and behavioral variable that may influence individuals’ assessments of alternative protein sources, sustainable food systems, and unconventional food options. Concerns about future access to sufficient and diverse food may lead individuals to reevaluate their traditional food preferences and adopt a more open attitude toward alternative sources, such as edible insects. However, this effect may not manifest directly, as the acceptance of edible insects depends not only on rational sustainability assessments but also on psychological mechanisms such as hesitation, disgust, curiosity, and interest toward new foods. Therefore, this study examines whether the perception of food insecurity is directly associated with gastronomy students’ intention to incorporate edible insects into recipes, as well as indirectly associated with it through food neophobia and food interest.

### 2.2. Protection Motivation Theory and Perceived Future Food Insecurity

PMT is not empirically tested as a complete theoretical model in this study. It is used only as a limited conceptual lens for framing perceived future food insecurity as a threat-related contextual perception, and none of the coping-appraisal components (response efficacy, self-efficacy, response cost) were directly measured.

PMT was developed by Rogers [19] to explain the relationship between fear-based messages and attitude change, and its scope was expanded in subsequent studies [18]. PMT posits two fundamental evaluation processes. Threat evaluation encompasses the perceived severity of a risk and the extent to which an individual perceives themselves as vulnerable to that risk. Coping evaluation, on the other hand, includes the belief that the recommended protective behavior will be effective (response efficacy) and the individual’s confidence in their ability to perform that behavior (self-efficacy), as well as the perceived costs of the behavior. The interaction between these two processes shapes protective motivation and, ultimately, adaptive behavior. Meta-analyses of PMT indicate that both the threat and coping components play a role in predicting protective behavior [20].

Future food insecurity can be conceptualized as a threat to the future. The form of food insecurity that is particularly prevalent in developed and middle-income societies is not so much absolute hunger as it is concern about potential future disruptions in access to nutritionally adequate and safe food. The literature on risk perception shows that individuals process threats not only in terms of objective probabilities but also through subjective evaluations [21]. Within this framework, the perception of a future inability to access safe protein is conceptualized as being associated with the threat assessment component of PMT, consistent with individuals evaluating alternative protein sources with high nutritional value.

In contrast, the current model is not a full-fledged PMT model and does not measure all dimensions of PMT. The study uses PMT as a guiding lens to address food insecurity as a threat-related contextual perception rather than as a causal trigger. Furthermore, it should be clearly emphasized that threat perception alone may not be sufficient. When the target behavior is culturally sensitive and emotionally aversive, the perception of threat may not translate directly into action. This reasoning suggests that the effect of perceived future food insecurity on the intention to incorporate edible insects into recipes may be weak or indirect. Nevertheless, it is theoretically defensible that a perceived threat to the food system could increase openness to resilient and resource-efficient protein alternatives. Based on this, the first hypothesis is proposed:

**H1.**  *Perceived future food insecurity positively and significantly predicts the intention to incorporate edible insects into recipes.*

### 2.3. Food Insecurity and Food Neophobia

Food neophobia is defined as a reluctance to try unfamiliar foods and is associated with uncertainty and anxiety regarding taste, texture, or appearance [13]. Neophobia is one of the strongest psychological barriers to the acceptance of edible insects; high levels of neophobia are associated with more negative attitudes toward insect-based products and a lower intention to try them [10,22]. Neophobia is not merely an individual fear; it is a behavioral pattern shaped by learning, experience, and culture, and can be reduced through exposure and repeated tasting experiences.

The relationship between perceived food insecurity and food neophobia may theoretically be bidirectional. On the one hand, the perception that access to food will be restricted in the future may reduce resistance to unfamiliar foods by making alternative and more accessible protein sources more instrumentally valuable. Studies examining scenarios of scarcity and hunger report that, under conditions of necessity, individuals’ willingness to consume unfamiliar foods increases, and small decreases in neophobia scores may be observed as hunger levels rise. On the other hand, it could also be argued that the perception of threat and risk may increase avoidance and perceived risk, which in turn could heighten neophobia. This two-way possibility makes the relationship theoretically intriguing. Within the current framework, the prevailing expectation is that food insecurity is expected to be negatively associated with neophobic resistance, consistent with a reinforced instrumental value attributed to functional protein sources:

**H2.**  *Perceived future food insecurity negatively predicts food neophobia.*

### 2.4. Food Insecurity and Food Interest

Food interest refers to an individual’s personal relationship with food, the importance they attribute to it, and their cognitive-emotional engagement with food-related activities. Interest is more broadly defined as an unobservable state of interest, arousal, and motivation triggered by a specific stimulus or situation [23]. In the context of food, this concept encompasses the level of psychological engagement and cognitive energy devoted to the entire life cycle of food, from its procurement to its preparation [24,25]. Engagement is influenced by dynamics such as risk, pleasure, and social consumption.

Under conditions of perceived food insecurity, individuals can be expected to pay closer attention to food sources, production systems, nutritional adequacy, and alternative ingredients. Individuals with a high level of food interest are more sensitive to vulnerabilities in food systems and evaluate the nutritional and environmental context of food more carefully. Therefore, the perception of food insecurity is expected to show a positive association with food interest, consistent with heightened cognitive salience of food. This sensitivity is expected to be particularly pronounced among gastronomy students, as food constitutes their academic and professional sphere of influence. In this regard:

**H3.**  *Perceived future food insecurity is a positive and significant predictor of food interest.*

### 2.5. Food Interest and Recipe Incorporation Intention

A high level of interest in food implies greater engagement with food knowledge, preparation, experimentation, and the creation of culinary meaning. This trait may strengthen the willingness to explore edible insects as an ingredient. Unlike personal consumption, applying edible insects in recipes requires not only a willingness to eat a particular food but also culinary creativity, technical curiosity, and professional openness. Highly interested individuals may be more inclined to incorporate these ingredients into menu development and recipe design processes, as they are able to grasp and rationalize the protein quality and environmental benefits of insects more quickly.

The relevant literature indicates that food interest and positive attitudes toward it are among the strongest predictors of acceptance of insect-based products; individuals with a high level of interest are more willing to try new and unfamiliar foods [5,7]. Studies conducted with student chefs show that culinary curiosity and a propensity for innovation increase openness to insect-based ingredients [17]. Accordingly,

**H4.**  *Food interest positively and significantly predicts the intention to incorporate edible insects into recipes.*

### 2.6. Food Neophobia and Recipe Incorporation Intention

Food neophobia is one of the most significant psychological barriers to the acceptance of edible insects. Insects are most commonly associated with disgust, fear of contamination, unfamiliar texture, cultural inappropriateness, and low sensory appeal [10,11,12]. Although the use of processed or concealed forms may reduce these barriers, neophobia remains an important factor [14]. It should be emphasized that, although culinary students possess technical knowledge and culinary competence, they are not immune to neophobic reactions; knowledge alone may not be sufficient to eliminate resistance based on disgust and unfamiliarity.

In the context of culinary applications, food neophobia is expected to be negatively associated not only with the desire to consume edible insects but also with the intention to process, prepare, and serve them to customers. Higher levels of food neophobia are expected to be associated with lower intention to apply edible insects, consistent with more limited experimentation in the kitchen. Accordingly,

**H5.**  *Food neophobia negatively and significantly predicts the intention to incorporate edible insects into recipes.*

### 2.7. The Mediating Roles of Food Neophobia and Food Interest

PMT does not predict that the perception of a threat will automatically translate into adaptive behavior; for the threat to become behaviorally meaningful, it is expected to be associated with a change in the individual’s psychological readiness. Since edible insects constitute a culturally sensitive and emotionally repulsive target behavior, it may not be expected that perceived food insecurity will directly lead to the intention to incorporate edible insects into recipes. Instead, the perception of threat may be linked to behavior through two distinct psychological channels.

Food neophobia and food interest are proposed as two distinct statistical mediators rather than causal mechanisms. Perceived future food insecurity is expected to be negatively associated with food neophobia and positively associated with food interest; in turn, food neophobia is expected to be negatively associated, and food interest positively associated, with recipe incorporation intention. These pathways represent resistance- and engagement-related correlational routes rather than opposing mechanisms.

In terms of mediation typology, situations where direct effects are not significant but indirect effects are significant are classified as “indirect-only mediation” [26]. This pattern is consistent with a mediation-based interpretation, in which the association between a threat to the food system and behavioral outcomes is expected to operate through underlying psychological pathways. The use of bootstrap-based methods is recommended for assessing mediation effects [27,28]. In line with this, the following two hypotheses are proposed:

**H6.**  *Food neophobia mediates the association between perceived future food insecurity and the intention to incorporate edible insects into recipes.*

**H7.**  *Food interest mediates the association between perceived future food insecurity and the intention to incorporate edible insects into recipes.*

**Figure 1:**
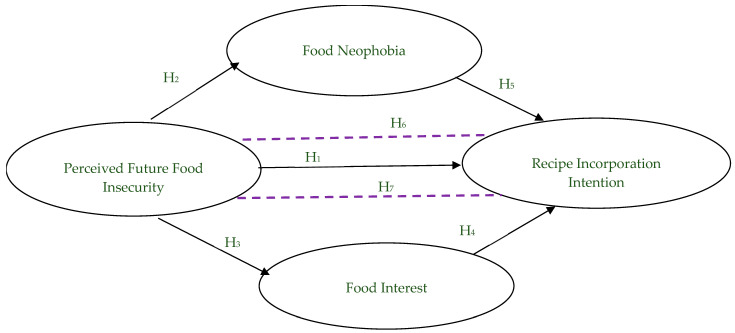
Proposed research model. Solid lines represent the hypothesized direct associations. H6 represents the indirect path PFFI → FN → RII, and H7 represents the indirect path PFFI → FI → RII. Source: Authors’ own elaboration.

Figure 1 illustrates the proposed research model, including the hypothesized direct and indirect relationships among perceived future food insecurity, food neophobia, food interest, and recipe incorporation intention.

## 3. Materials and Methods

### 3.1. Study Design and Participants

A cross-sectional survey design was used in this study. The study population consists of undergraduate students enrolled in Gastronomy and Culinary Arts programs at public universities in Turkey. The theoretical rationale for selecting this population is that gastronomy students may play a pivotal role in the future, as chefs, menu developers, culinary educators, entrepreneurs, or food and beverage managers, in integrating alternative protein sources into culinary practices. During the data collection process, the maximum diversity sampling method (one of the purposeful sampling techniques) was used; the goal was to reach participants from all seven geographic regions of Turkey [29]. Thus, the aim was to ensure that the sample reflected the evaluations of students who had grown up in different socio-cultural environments and possessed diverse gastronomic experiences. This purposive maximum-variation strategy was pursued by distributing the survey through faculty contacts across the seven regions; however, because participation depended on students’ voluntary response to an online survey link, the final sample also involved convenience and self-selection components and should not be considered representative in a probabilistic sense.

A total of 400 valid questionnaires were obtained for use in the analyses. According to relevant statistics, the number of students enrolled in Gastronomy and Culinary Arts undergraduate programs across Turkey is estimated to be in the range of approximately 10,000–13,000. According to the sample size table presented by Bartlett, Kotrlik, and Higgins [30], the recommended minimum sample size for a population of 10,000 at a 95% confidence level and a 5% margin of error is 370. The 400 valid surveys obtained exceed this threshold and were deemed sufficient for the planned analyses. This population-based benchmark was used only as a general reference for the adequacy of the sample size and should not be interpreted as establishing a formal sampling margin of error under the non-probability design actually used. However, since a non-probability sampling strategy with convenience and self-selection characteristics was used, the generalizability of the findings should be interpreted with caution.

During the data collection process, the goal was to reach participants from Turkey’s seven geographic regions, and all regions were represented in the final sample. The survey link was distributed to students through faculty members at the relevant universities. Since all scale items were mandatory, there were no missing values in the dataset. Because a non-probability sampling strategy was used, the generalizability of the findings is limited. Potential control variables such as previous experience with edible insects (tasting or eating), enrollment in a sustainable gastronomy course, and dietary orientation were not collected; this issue is addressed in the limitations section.

**Figure 2:**
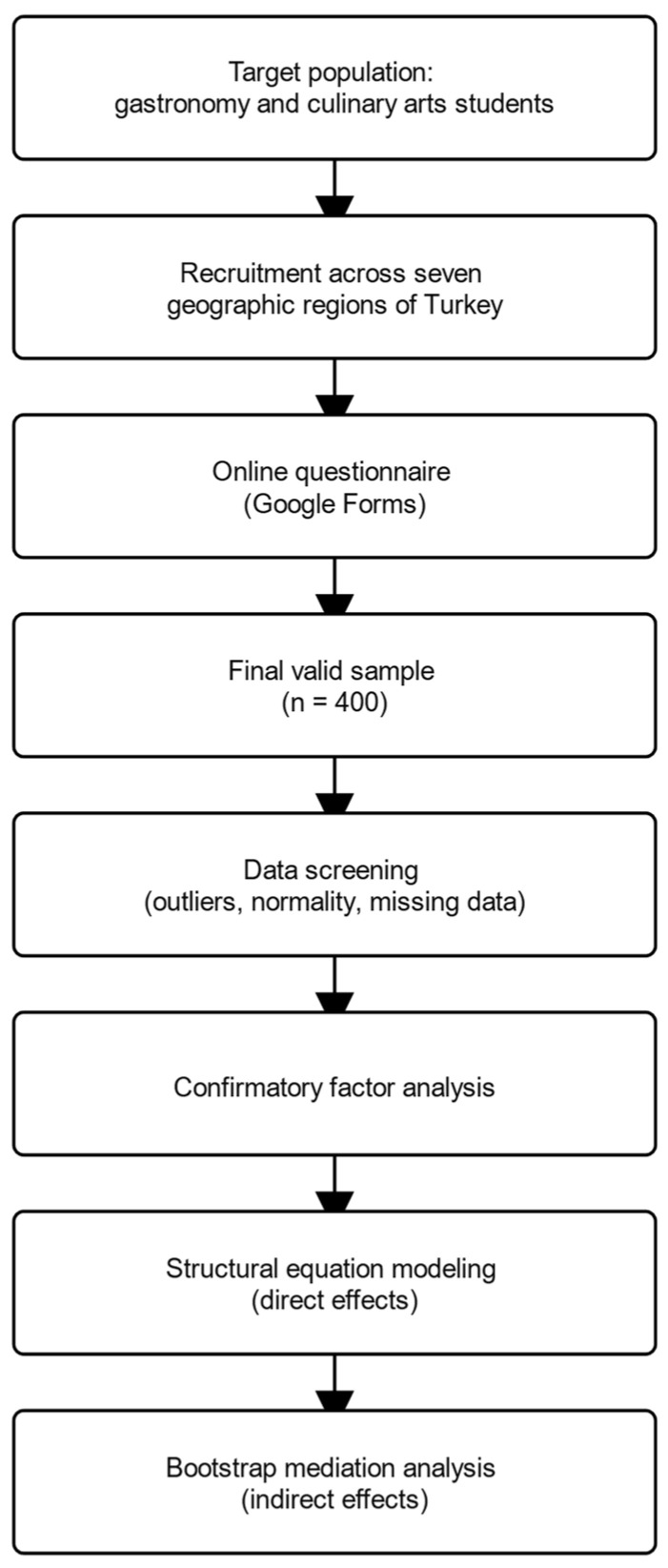
Overview of the research process. Authors’ own Elaboration.

Figure 2 summarizes the research process, from target population definition to the bootstrap mediation analysis.

### 3.2. The Data Collection Process and Ethics

The data for this study were collected via an online survey between 1 February 2026, and 31 March 2026. The survey form was created using Google Forms and distributed to students with the support of faculty members at the relevant universities. Upon accessing the form, participants were first informed about the study’s purpose, scope, and ethical principles; it was emphasized that participation was voluntary, and informed consent was obtained. Since all scale items were defined as mandatory, there were no missing values in the dataset. The study was reviewed by the Ethics Committee for Social and Humanities Sciences at Recep Tayyip Erdoğan University and was deemed ethically sound by Decision No. 2026/47 dated 28 January 2026.

### 3.3. Measurement Instruments

The survey form includes demographic questions (gender, educational level, and geographic region of residence) along with statements related to four latent constructs. Food insecurity was treated as the independent variable, the intention to incorporate edible insects into recipes as the dependent variable, and food neophobia and food interest as mediating variables. Food insecurity was measured using a 9-item scale developed by Villagómez-Ornelas et al. [31]; food neophobia was measured using the 10-item Food Neophobia Scale developed by Pliner and Hobden [13]; the intention to incorporate edible insects into recipes was measured using a 12-item scale developed by Cicatiello et al. [32]; and food interest was measured using a 3-item scale adapted into Turkish by Onat [33] and based on the studies by Leong et al. [34] and Mittal [25]. All items were rated using a 5-point Likert-type scale (1 = Strongly disagree, 5 = Strongly agree).

The processes of using and adapting scales in Turkish vary depending on the sources of the variables. For the food neophobia scale, the original scale developed by Pliner and Hobden [13] was used with the Turkish wording employed by Karakuş, Onat, and Sarıgül Yılmaz [35]. For the Food Interest Scale, the items used in the Turkish literature by Onat [33], which are based on the studies by Leong et al. [34] and Mittal [25], were adopted; these items were adapted to fit the context of edible insects and recipe application in this study. The scales for food insecurity and the intention to incorporate edible insects into recipes were translated into Turkish based on the items in the original sources. During the translation process, input was sought from academics proficient in foreign languages and from experts in the field of gastronomy and food and beverage (three academics proficient in English and two scholars specializing in gastronomy and food and beverage management, reviewing independently and discussing disagreements until consensus was reached); the linguistic clarity, conceptual equivalence, and suitability of the items for the professional context of gastronomy students were evaluated.

In particular, the items regarding the intention to incorporate edible insects into recipes have been adapted to reflect culinary practices, such as adding them to the menu, preparing, cooking, and serving, in a way that distinguishes them from the intention for personal consumption. The adapted outcome measure was intended to capture professional culinary implementation rather than personal consumption; its items referred to preparing, cooking, serving, and incorporating insect-derived ingredients into recipes or menus. This adaptation is intended to capture professional implementation rather than fully establishing recipe incorporation intention as a distinct behavioral construct, and the translation review was qualitative and consensus-based among the bilingual academics and gastronomy experts described below; no formal content-validity index was calculated. Participants were not instructed to consider a specific insect species or product format; they evaluated edible insects as a general food ingredient category, and their responses should be interpreted accordingly. A formal back-translation procedure and a separate pilot study were not conducted. This limitation should be considered when interpreting the linguistic and contextual equivalence of the adapted measures. Consultation with bilingual academics and gastronomy experts provided a procedural safeguard for linguistic clarity, conceptual equivalence, and contextual appropriateness.

### 3.4. Data Preparation and Analysis Strategy

All statistical analyses were performed using IBM SPSS Statistics version 26 (IBM Corp., Armonk, NY, USA) and IBM SPSS AMOS version 24 (IBM Corp., Chicago, IL, USA). Since the Food Neophobia Scale contains both positive and negative statements, the positive statements (GN1, GN2, GN4, GN5, GN6, and GN7) were reverse-coded to ensure that all items on the scale measure the same construct. Following reverse coding, higher scores indicate higher food neophobia (stronger reluctance toward new foods). Since responses were mandatory and there were no missing values in the dataset, no missing data handling was required. Mahalanobis distances were examined to assess multivariate outliers; since no observations were found to exceed the critical threshold, no survey data were excluded from any dataset [36]. Skewness and kurtosis values were used to assess univariate normality; the fact that these values fell within the recommended ±1.96 limits was deemed to support the use of parametric structural equation modeling procedures [37].

The measurement model was tested using confirmatory factor analysis (CFA). Reliability was assessed using Cronbach’s alpha and composite reliability (CR); convergent validity was assessed using average variance extracted (AVE) [38]; and discriminant validity was assessed using the heterotrait–monotrait (HTMT) ratio [39]. Direct effects were tested using structural equation modeling (SEM); indirect effects were estimated using a bias-corrected bootstrap method with 1000 resamples and a 95% confidence interval [28,40]. The typology proposed by Zhao, Lynch, and Chen [26] was used as the basis for interpreting the mediating effects. Model fit was assessed using the following conventional criteria: χ^2^/df ≤ 5.00, RMSEA ≤ 0.08, SRMR ≤ 0.08, CFI and TLI ≥ 0.90, and GFI and AGFI ≥ 0.80 [41,42,43].

Since the data were collected from the same participants via a single survey, at a single point in time, the risk of common method bias (CMB) was assessed. To this end, Harman’s one-factor test and the VIF analysis for full collinearity proposed by Kock [44] were applied; the relevant results are reported in the Findings section. Additionally, the proposed partial mediation model was compared with the full mediation model (in which the direct path is removed) using the nested chi-square difference and AIC criteria. A supplementary gender-based multigroup comparison was considered; because a defensible comparison requires formally tested measurement invariance rather than item-level loading comparisons alone, and because previous insect exposure was not measured, this exploratory analysis is not reported in the main text (see Section 5.3, Limitations and Future Research).

## 4. Findings

### 4.1. Participant Profile

**Figure 3:**
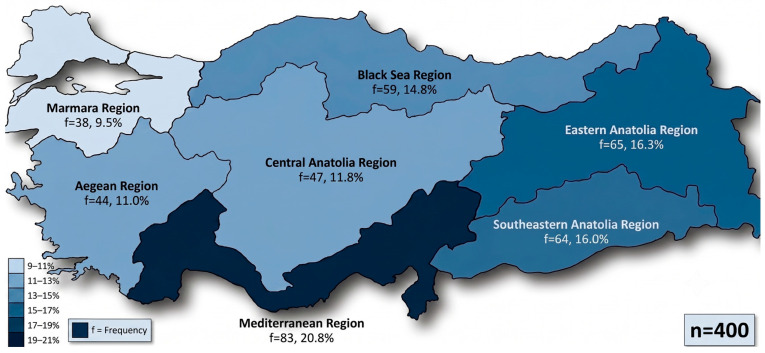
Distribution of Participants by Geographic Region Source: Authors’ elaboration.

A total of 64% of the participants (n = 256) were women, and 36% (n = 144) were men. This distribution is consistent with the overall student profile of the Gastronomy and Culinary Arts programs. In terms of class level, the sample is evenly distributed: 28.5% are fourth-year students (n = 114), 27% are first-year students (n = 108), 24% are second-year students (n = 96), and 20.5% are third-year students (n = 82). In terms of geographic region distribution, the highest participation came from the Mediterranean Region; participants were recruited from all seven regions. Participant characteristics are presented in Figure 3.

### 4.2. Preliminary Analyses

Mahalanobis distances were examined to identify multivariate outliers, and no observations were found to exceed the critical threshold. In the assessment of univariate normality, it was observed that the skewness values for all variables ranged from −1.091 to 1.562, while the kurtosis values ranged from −1.171 to 1.718. The fact that these values fall within the recommended ±1.96 limits supports the use of parametric analysis techniques [44].

### 4.3. Measurement Model

The initial measurement model was based on previously developed instruments, after which the adapted item structure was evaluated through CFA. In the initial measurement model, several items showed standardized factor loadings below 0.50 and/or localized model-fit problems. Accordingly, while maintaining theoretical consistency, items GN3, GN7, GN8, GN9, and GN10 from the Food Neophobia Scale, and items YB7, YB8, YB9, YB10, YB11, and YB12 from the Intention to Incorporate Edible Insects into Recipes Scale were sequentially removed from the analysis. After each item was removed, the CFA was repeated; all retained items showed acceptable standardized loadings, and the overall model fit improved.

The composite reliability (CR) values ranged from 0.83 to 0.96, the AVE values from 0.50 to 0.80, and the Cronbach’s alpha values from 0.834 to 0.961. All CR values were above 0.70 and all AVE values were above 0.50, indicating that construct reliability and convergent validity were established [44]. The fit indices of the measurement model (χ^2^/df = 3.115; AGFI = 0.841; GFI = 0.873; CFI = 0.929; TLI = 0.919; RMSEA = 0.073; SRMR = 0.072) are within acceptable limits. Standardized factor loadings and reliability indices are presented in Table 1.

Discriminant validity was assessed using the heterotrait–monotrait (HTMT) ratio. The HTMT values are presented in Table 2; all values fell below the 0.90 threshold and the stricter 0.85 threshold. The highest HTMT value was observed between food interest and recipe incorporation intention (HTMT = 0.788), which is still below the threshold values. This result indicates that discriminant validity is established despite the high correlation (r = 0.720) between the two constructs.

Common method bias (CMB) was assessed using two methods. In Harman’s one-factor test, the unrotated first component explained only 32.13% of the total variance, which fell below the 50% threshold [45,46]. In the VIF analysis for full collinearity, the VIF values for all constructs were below the 3.3 threshold (food insecurity = 1.06; food neophobia = 1.18; food interest = 2.08; recipe incorporation intention = 2.19). These findings indicate that the likelihood of common method bias significantly distorting the results is low [43]. Nevertheless, common method bias cannot be completely ruled out, as all variables were collected from the same respondents at a single point in time.

Several items were removed from the measurement model due to low standardization load or redundancy. Since most of the removed items were negative/avoidance (barrier)-oriented statements, the retained scales should be interpreted as context-specific abbreviated forms. In the food neophobia scale, items that were predominantly reverse-coded (measuring openness to new/different cuisines) have been retained, while in the Intention to Incorporate Edible Insects into Recipes Scale, items primarily reflecting the approach dimension (willingness to incorporate) have been retained; this indicates a narrowing of the construct’s scope and is discussed in the limitations section. More specifically, the five Food Neophobia Scale items removed during CFA (GN3, GN7, GN8, GN9, GN10) directly worded distrust, refusal, and pickiness toward unfamiliar food in general (e.g., reluctance to eat foods not previously tried, not trusting new foods, refusing food that is unknown or unfamiliar), whereas the five retained items (GN1, GN2, GN4, GN5, GN6) are worded around openness to different ethnic and international cuisines specifically (e.g., enjoying ethnic restaurants, trying foods from other countries). The item-removal pattern therefore narrowed the Food Neophobia Scale by reducing the representation of general food distrust and refusal, leaving a retained version that more narrowly reflects openness to foreign or unfamiliar cuisines rather than food neophobia in its broadest sense. Similarly, the six removed items from the Intention to Incorporate Edible Insects into Recipes Scale (YB7–YB12) were worded around customer-facing and sensory barriers to service (cultural fit with the kitchen’s cuisine, unappealing presentation, unpleasant texture, uncertain flavor pairing, fear of serving insect-themed dishes to guests, and hygiene concerns), whereas the six retained items (YB1–YB6) express personal willingness to prepare, cook, or add insects to a menu out of curiosity or nutritional interest. The removal of these items therefore reduced the representation of guest-facing service concerns and safety/sensory barriers, leaving a stronger emphasis on personal exploratory willingness. The retained scale should accordingly be interpreted as a narrower measure of recipe incorporation intention than the original instrument, focused on personal willingness to experiment rather than the full scope of professional culinary adoption, including its customer-facing dimension. Because item deletion was partly guided by model-fit considerations, the improved fit indices may also partially reflect post hoc model refinement; replication with the complete scales, or with independently validated abbreviated forms, is therefore necessary.

### 4.4. Descriptive Statistics and Correlations

The means, standard deviations, and correlation coefficients for the variables are presented in Table 3. There was a significant negative relationship between perceived future food insecurity and food neophobia (r = −0.227; *p* < 0.01); while low-level, statistically significant positive relationships were found between food insecurity and recipe incorporation intention (r = 0.108; *p* < 0.05) and food interest (r = 0.106; *p* < 0.05). Significant negative correlations were observed between food neophobia and recipe incorporation intention (r = −0.344; *p* < 0.01) and food interest (r = −0.263; *p* < 0.01). The highest correlation was found between food interest and recipe incorporation intention (r = 0.720; *p* < 0.01). This strong relationship is theoretically expected; however, since it also raises the possibility of measurement overlap, discriminant validity was examined using the HTMT ratio, and the item contents of the two constructs were conceptually compared (see Section 4.3 and Limitations).

### 4.5. Structural Model and Hypothesis Tests

The results of the structural equation modeling regarding direct effects are presented in Table 4. The direct effect of perceived future food insecurity on recipe incorporation intention was not found to be significant (β = −0.015; *t* = −0.352; *p* = 0.725); therefore, H1 was not supported. In contrast, food insecurity predicted food neophobia negatively, with a statistically significant but relatively small effect (β = −0.212; *t* = −3.527; *p* < 0.001); and food interest with a positive, statistically significant but weak effect (β = 0.138; *t* = 2.401; *p* < 0.05). Thus, H2 and H3 were supported. Food interest has a strong effect on recipe incorporation intention (β = 0.715; *t* = 14.196; *p* < 0.001), which supports H4. Food neophobia, on the other hand, predicted recipe incorporation intention negatively with a relatively small effect (β = −0.225; *t* = −4.932; *p* < 0.001), supporting H5. The explained variance ratios are 0.019 for food interest, 0.045 for food neophobia, and 0.568 for recipe incorporation intention. Perceived future food insecurity explained only 1.9% of the variance in food interest and 4.5% of the variance in food neophobia, indicating very limited predictive relevance for the two mediators. The comparatively high explained variance in recipe incorporation intention primarily reflects the strong associations of food interest and food neophobia with the outcome rather than a substantial explanatory contribution from perceived future food insecurity. The model fit indices are within acceptable limits (χ^2^/df = 3.194; AGFI = 0.838; GFI = 0.870; CFI = 0.926; TLI = 0.916; RMSEA = 0.074; SRMR = 0.080).

To assess the robustness of the proposed partial mediation model (which includes a direct path from food insecurity to recipe incorporation intention), a comparison was made with a full mediation model in which this path was removed. In the nested chi-square difference test, removing the direct path did not significantly worsen model fit (Δχ^2^(1) = 0.494; *p* = 0.482); furthermore, the AIC value of the full mediation model (94.77) was found to be lower than that of the partial mediation model (96.77). The fact that the more parsimonious full mediation model is statistically equivalent to (and slightly preferred over) the partial mediation model supports the interpretation that the effect of food insecurity on recipe incorporation intention operates indirectly rather than directly.

Note: The primary CFA and SEM analyses were conducted using AMOS 24. HTMT, common-method bias identification, and nested model comparisons were performed using supplementary statistical calculations as robustness checks.

### 4.6. Mediation Analysis

The bootstrap results regarding indirect effects are presented in Table 5. A significant mediating role of food neophobia was found in the effect of perceived future food insecurity on recipe incorporation intention (indirect effect = 0.079; *p* = 0.040; 95% CI [0.028, 0.120]). Similarly, food interest also played a significant mediating role (indirect effect = 0.098; *p* = 0.024; 95% CI [0.036, 0.193]). Since the confidence intervals for both pathways do not include zero, the indirect effects are significant; in contrast, the direct effect of food insecurity on recipe incorporation intention is not significant. According to the typology of Zhao, Lynch, and Chen [26], this pattern represents only indirect mediation for both pathways. Therefore, H6 and H7 are supported.

## 5. Discussion

This study shows that perceived future food insecurity is not directly related to gastronomy students’ intention to incorporate edible insects into recipes; rather, this connection emerges through indirect pathways. Perceived food insecurity was found to be associated with lower food neophobia and higher food interest. Food interest is strongly associated with the intention to incorporate edible insects into recipes, while food neophobia is negatively associated with it. Both mediators indirectly link perceived future food insecurity to recipe incorporation intention. Taken as a whole, the findings reveal a psychologically mediated pattern regarding the culinary adoption of edible insects. This pattern suggests that threat-related perceptions alone are insufficient and that their association with recipe incorporation intention is statistically linked to food neophobia and food interest.

One notable finding was the absence of a significant direct association between perceived future food insecurity and recipe incorporation intention. While some studies suggest that concerns about sustainability, the environment, or food security may increase openness to alternative proteins [4,5], acceptance of edible insects continues to be limited by disgust, neophobia, cultural distance, unfamiliarity, safety concerns, and sensory expectations [7,10,12,14,22]. Systematic reviews indicate that insects are the least accepted option among alternative proteins [5].

This pattern is consistent with a prediction of PMT: the perception of a threat does not automatically lead to adaptive behavior. When the target behavior is culturally sensitive and emotionally charged, the threat alone is insufficient to trigger action. Therefore, the fact that the direct effect of perceived future food insecurity proved to be insignificant should be interpreted within the broader mediational framework of the study rather than as evidence against the hypothesized model. A macro-level threat to the food system appears to be associated with behavioral outcomes primarily through its association with an individual’s psychological readiness (i.e., lower food neophobia and higher food interest). Thus, the findings are broadly consistent with the PMT proposition that threat perception alone may be insufficient to produce adaptive behavioral intention, with statistically significant indirect associations observed through food neophobia and food interest in this context. It should be emphasized that this interpretation is not causal, as it is based on a cross-sectional dataset; the findings should be interpreted not as a transformation but as a theoretically consistent pattern of relationships. Because coping-appraisal components were not directly measured, the study cannot determine which PMT coping mechanisms account for this pattern.

H2 is supported; perceived future food insecurity negatively predicted food neophobia. This finding is consistent with the interpretation that the perception of future restrictions on food access is associated with reduced resistance to functional but unfamiliar protein sources. Studies examining scarcity and hunger scenarios report that conditions of necessity can increase the desire for unfamiliar foods and that small decreases in neophobia scores may be observed as hunger levels rise. This supports the idea that scarcity-based instrumental value attribution can mitigate neophobic resistance.

However, there is also an alternative line of reasoning: stress and the perception of risk can increase avoidance and perceived danger, which in turn could heighten neophobia. The present findings do not support this alternative pathway; instead, the negative direction of the observed relationship suggests that perceived food insecurity is associated with reduced neophobia toward insects, consistent with the possibility that perceived future scarcity is associated with greater openness to unfamiliar protein sources. Nevertheless, the low effect size and explained variance serve as a reminder that food insecurity is not the sole determinant of neophobia; factors such as personality, culture, and past experiences also play a role.

H3 was supported; perceived future food insecurity positively predicted food interest at a weak level. This finding is consistent with the interpretation that the perception of food insecurity may increase cognitive awareness of food systems, nutritional adequacy, and alternative ingredients. Since food is an academic and professional domain for gastronomy students, one might expect this sensitivity to be more pronounced. However, the low explained variance (R^2^ = 0.019) indicates that food insecurity is not the primary determinant of engagement. Factors such as culinary identity, prior exposure, sustainability education, and innovation orientation may play a stronger role in shaping food interest. Therefore, this finding should be interpreted cautiously, as a weak contextual association.

Statistical significance should not be confused with practical explanatory strength. Perceived future food insecurity accounted for only 1.9% of the variance in food interest and 4.5% of the variance in food neophobia. These values indicate that perceived future food insecurity is a statistically detectable but weak contextual correlate rather than a major determinant of either mediator, and the findings should not be interpreted as evidence that food-system threat perceptions substantially shape culinary interest or neophobic responses.

H4 is the study’s strongest and, from a practical standpoint, most central finding: food interest strongly predicted recipe incorporation intention (β = 0.715). This result indicates that following recipes is not merely a matter of acceptance; it requires curiosity, professional interest, engagement with ingredients, and culinary experimentation. Individuals with high levels of interest are more likely to quickly grasp the nutritional and environmental benefits of insects and incorporate them into menu development and recipe design processes [7]. Studies conducted with student chefs also reveal that culinary curiosity and a propensity for innovation increase openness to insect-based ingredients; in particular, they enhance the feasibility of processed forms [17]. This finding highlights the role of culinary education as a catalyst in normalizing insect-based ingredients. However, while the HTMT results support discriminant validity, the strong relationship between food interest and recipe incorporation intention suggests that future studies should examine whether these constructs remain distinct when behavioral or experimental measures of culinary adoption are used.

H5 was supported; food neophobia negatively predicted recipe incorporation intention. This finding is consistent with the literature showing that neophobia is intertwined with disgust toward insect-based products, unfamiliarity, sensory barriers, cultural norms, and perceptions of safety [10,11,22]. An important point is that, despite possessing technical knowledge and culinary proficiency, culinary students are not immune to neophobic reactions; knowledge alone is not sufficient to eliminate disgust-based resistance. However, the use of insects in processed or invisible forms (such as flour or protein powder) can reduce this resistance [14]; this suggests a concrete strategy for practical application.

When H6 and H7 are evaluated together, the theoretical core of the study becomes clear. Food neophobia and food interest mediated the effect of perceived future food insecurity on recipe incorporation intention only indirectly. PMT does not suggest that a threat automatically elicits adaptive behavior; a perceived threat becomes behaviorally meaningful only when it alters the individual’s psychological readiness. In this study, the threat of food insecurity was linked to intention through two distinct psychological channels: (a) reducing food neophobia to remove psychological resistance; (b) increasing food interest to strengthen cognitive and professional involvement.

The fact that the direct effect is insignificant while the indirect effect is significant indicates only indirect mediation, according to the typology proposed by Zhao et al. [26]. This pattern is theoretically consistent with the absence of a direct effect, as culturally sensitive target behaviors are more likely to be associated with macro-level threats indirectly, through psychological mediating pathways. Thus, the study examines a context-specific mediation structure in which perceived future food insecurity is associated with food neophobia and food interest, which in turn are associated with recipe incorporation intention; this should be regarded as a preliminary, exploratory pattern rather than a new theoretical model. The observed coefficients may also partly reflect omitted-variable effects, since prior exposure, tasting experience, sustainability education, dietary orientation, disgust sensitivity, and general food familiarity were not included in the model. Previous exposure to edible insects, tasting experience, familiarity, sustainability education, dietary orientation, and disgust sensitivity are likely to be more proximal predictors of insect-related acceptance than generalized perceptions of future food insecurity; their omission limits the explanatory completeness of the model and may have affected the estimated structural relationships. The model should therefore be interpreted as a restricted explanatory model rather than a comprehensive account of culinary adoption. From a food systems perspective, this finding suggests that the integration of edible insects into sustainable and circular food systems is related not only to technical production capacity but also to the capacity of culinary professionals to adapt these ingredients into acceptable recipe and menu formats. Although this was not directly tested in the present study, it is plausible that reaching consumers with the produced biomass may be more feasible through culinary normalization and processed or low-visibility product formats [47].

The findings may also be interpreted alongside planned behavior and novel food acceptance research, which emphasizes attitudes, perceived control, familiarity, and social norms. However, these constructs were not measured in the present study, and therefore such comparisons remain interpretive rather than empirical.

### 5.1. Theoretical Contributions

The study’s contribution is primarily contextual rather than theory-building. First, it examines gastronomy students, an understudied professional-training group in the edible-insect literature, rather than a general consumer sample. Second, it distinguishes recipe incorporation intention from personal consumption intention by focusing on preparation, cooking, serving, and menu integration, and treats it as a separate outcome rather than an extension of eating intention. Third, it documents context-specific indirect associations between perceived future food insecurity, food neophobia, food interest, and recipe incorporation intention within a single cross-sectional sample. Because PMT was not fully operationalized and its coping-appraisal components were not measured, these findings should not be interpreted as an extension or empirical test of PMT, but as an application of PMT-inspired framing to a new professional-behavioral context.

### 5.2. Implications for Practice

The findings offer concrete, multi-layered implications for the culinary adoption of edible insects. The following recommendations are organized into implications directly supported by the present findings and broader implications informed by previous literature, so that the two are not presented with the same evidentiary weight.

#### Implications Directly Supported by the Present Findings

For culinary education: Since food interest is the strongest predictor of recipe incorporation intention, edible insect modules can be added to sustainable gastronomy courses, and curricula should give particular attention to cultivating food interest and engagement; because food neophobia is negatively associated with recipe incorporation intention, curricula should also address unfamiliarity with insect-based ingredients. The present study did not test specific interventions such as workshops, tasting sessions, sensory training, product-format sequencing, or repeated-exposure designs; these are discussed below as broader, literature-informed recommendations rather than findings of this study.

Broader Implications Informed by Previous Literature: The following recommendations extend beyond the variables directly tested in the present study and are informed by previous literature rather than by the present findings alone.

For culinary education (literature-informed): Previous research suggests that workshops on insect ingredients, controlled tasting and sensory training sessions, and recipe development projects may be useful curricular tools; starting with low-visibility product forms (flour, protein powder) and deepening exposure gradually is also suggested by prior work as a way to ease neophobic resistance [14,17]; these specific interventions were not tested in the present study and their effectiveness for this population remains to be demonstrated.

For food and beverage businesses: Previous research suggests that insect-based products in less conspicuous forms, such as bread, pasta, crackers, and snacks, may serve as an initial strategy to boost acceptance; this was not directly tested with product-format comparisons in the present study. Regardless of menu framing, insect content must always be clearly disclosed to consumers as a mandatory ingredient and allergen information; within this requirement, small-portion tasting menus emphasizing sustainable protein and nutritional value, along with chef-led explanations and storytelling, can help ease initial acceptance [48].

To build consumer trust: Transparently sharing information on sourcing, production, and hygiene, along with education on allergenicity and microbiological safety can support consumer confidence and, consequently, acceptance [8,9].

For policy and the food system: Programs that promote alternative protein literacy, safety and regulatory standards, university–industry collaborations, and recipe development tailored to local gastronomy may help facilitate the integration of insects into circular food systems, based on the broader literature rather than on a policy-level test within this study. These implications should be read as preliminary and context-specific, reflecting the intention-level findings of a single cross-sectional sample of gastronomy students rather than a validated policy or business framework.

### 5.3. Limitations and Future Research

This study has several limitations. First, the cross-sectional design limits causal inferences; the relationships are predictive in nature and do not provide evidence of cause and effect. Second, because the sample was recruited through faculty networks and voluntary online participation, it should be considered a non-probability sample with convenience and self-selection characteristics; representation across all seven regions does not establish national representativeness, and non-probability sampling limits generalizability. Third, self-report data may be subject to social desirability bias. Fourth, not all dimensions of PMT (perceived severity, vulnerability, response efficacy, self-efficacy, and response cost) were measured; therefore, the model is not a full PMT test but rather a PMT-informed framework. Fifth, the dependent variable measures intention rather than actual recipe incorporation behavior (actual cooking, tasting, or adding to the menu). Sixth, potential control variables, such as participants’ prior exposure to edible insects (hearing about or tasting them), participation in a sustainable gastronomy course, and dietary orientation were not collected; this is a significant limitation, as these variables could influence psychological constructs.

Seventh, a large number of items were removed from the Food Neophobia Scale and the Recipe Incorporation Intention Scale during the CFA process; these omissions may have narrowed the construct’s scope (specifically, the avoidance dimension in neophobia and the negative/avoidance dimension in recipe incorporation intention have been weakened). Therefore, the retained scales should be interpreted as context-specific abbreviated forms and revalidated in different culinary education samples. Eighth, the study did not distinguish between insect species; acceptance may vary depending on the species (e.g., mealworms, house crickets, grasshoppers). Ninth, no distinction was made regarding product format; forms such as whole insects, insect flour, and protein powder were not evaluated separately. Tenth, the cultural context is limited to Turkey; results may differ in countries with a tradition of entomophagy. Eleventh, although the HTMT value remained below conventional thresholds, the strong association between food interest and recipe incorporation intention raises the possibility of partial measurement overlap. The lack of distinction by species and product form is an important limitation that should be particularly noted in the context of the edible insect literature.

Future research could overcome these limitations through experimental and longitudinal designs, actual taste tests, cross-cultural comparisons, hands-on cooking tasks, intervention studies, and comparisons of product formats. Furthermore, given the low explanatory power of food insecurity on interest and neophobia, it is recommended that additional variables, such as culinary identity, prior exposure, and sustainability education, be included in the model. A gender-based multigroup comparison was considered but is not reported here, because a defensible comparison requires acceptable configural fit and formally tested measurement invariance (equality-constrained unstandardized loadings compared against a freely estimated model, with ΔCFI, ΔRMSEA, and Δχ^2^ reported) rather than separate item-level loading comparisons, and because previous insect exposure—arguably the more theoretically relevant grouping variable—was not collected. Future studies with the primary analysis software and a formally specified invariance-testing procedure could examine gender and exposure-based group differences.

## 6. Conclusions

This study aimed to examine the direct and indirect associations of perceived future food insecurity with gastronomy students’ intention to incorporate edible insects into recipes, through the mediating variables of food neophobia and food interest. Protection Motivation Theory was used as a guiding framework; confirmatory factor analysis and structural equation modeling were applied in a cross-sectional study conducted with 400 culinary students from seven geographic regions of Turkey.

The key finding is that perceived future food insecurity is associated with recipe incorporation intention not directly, but through indirect channels. Perceived future food insecurity was found to be associated with lower food neophobia and higher food interest; food interest was strongly associated with recipe incorporation intention, while food neophobia was negatively associated with it. Both specific indirect associations were statistically significant, whereas the direct association between perceived future food insecurity and recipe incorporation intention was not significant. This pattern suggests that a threat to the food system alone is not sufficient; it is associated with recipe incorporation intention through statistically significant indirect associations involving lower food neophobia and higher food interest.

This finding underscores the importance of culinary education. The fact that food interest is the strongest predictor of recipe incorporation intention indicates that the professional and cognitive engagement of future culinary professionals with insect-based ingredients is an important factor in incorporating these ingredients into practice. Education should prepare students not only to taste insects but also to transform them into palatable recipes.

In conclusion, this study examines a context-specific mediation structure in which food neophobia and food interest are associated with gastronomy students’ recipe incorporation intention regarding edible insects, alongside perceived future food insecurity. Perceived future food insecurity was not directly associated with recipe incorporation intention; it was associated with recipe incorporation intention only indirectly, through lower food neophobia and higher food interest, and it explained only a small proportion of the variance in both mediators (1.9% for food interest and 4.5% for food neophobia). The findings should therefore be interpreted as preliminary, context-specific evidence rather than as a comprehensive explanatory model of culinary adoption, and as documenting statistically significant indirect associations rather than establishing a new theoretical model. The integration of edible insects into circular and sustainable food systems is likely to be associated not only with perceived future food insecurity but, more importantly, with the extent to which future culinary professionals are psychologically and professionally engaged with insect-based ingredients.

## Figures and Tables

**Table 1:** Measurement model: standardized factor loadings, CR, AVE, and Cronbach’s alpha.

Dimension	Item	Std. Load	*t*	CR	AVE	α
Perceived Future Food Insecurity	GG1	0.594	10.564	0.90	0.50	0.901
	GG2	0.647	11.400			
	GG3	0.687	12.015			
	GG4	0.775	13.264			
	GG5	0.756	13.001			
	GG6	0.778	13.314			
	GG7	0.694	12.079			
	GG8	0.727	12.575			
	GG9	0.661	Fixed			
Food Neophobia	GN1	0.566	Fixed	0.83	0.50	0.838
	GN2	0.608	12.283			
	GN4	0.781	10.759			
	GN5	0.847	11.065			
	GN6	0.679	9.958			
Food Interest	Gİ1	0.833	Fixed	0.85	0.66	0.834
	Gİ2	0.831	18.011			
	Gİ3	0.762	16.337			
Intention to Incorporate Edible Insects into Recipes	YB1	0.897	Fixed	0.96	0.80	0.961
	YB2	0.925	30.049			
	YB3	0.905	28.400			
	YB4	0.890	27.238			
	YB5	0.866	25.505			
	YB6	0.865	25.431			

Note. GG: perceived future food insecurity; GN: food neophobia; Gİ: food interest; YB: intention to incorporate edible insects into recipes. Fit indices: χ^2^/df = 3.115; AGFI = 0.841; CFI = 0.929; RMSEA = 0.073; GFI = 0.873; SRMR = 0.072; TLI = 0.919. “Fixed” = item fixed to reference loading of 1 (no standard error or significance test). Discriminant validity was assessed using the heterotrait–monotrait (HTMT) ratio (see Table 2). Authors’ own Elaboration.

**Table 2:** Discriminant validity: HTMT ratios.

Structure	1	2	3	4
1. Perceived Future Food Insecurity	-			
2. Food Neophobia	0.266	-		
3. Recipe Incorporation Intention	0.116	0.381	-	
4. Food Interest	0.125	0.304	0.788	-

Note: All HTMT values are below the 0.85 and 0.90 thresholds; discriminant validity is supported. Authors’ own Elaboration.

**Table 3:** Descriptive statistics and correlations.

Variable	Mean	SD	1	2	3	4
1. Perceived Future Food Insecurity	3.642	0.808	1			
2. Food Neophobia	2.301	0.813	−0.227 **	1		
3. Recipe Incorporation Intention	2.284	1.190	0.108 *	−0.344 **	1	
4. Food Interest	1.994	1.051	0.106 *	−0.263 **	0.720 **	1

Note: N = 400. * *p* < 0.05; ** *p* < 0.01 (two-tailed). Authors’ own Elaboration.

**Table 4:** Structural model: direct effects.

Hypothesis	Relationship	β	*t*	*p*	Result
H1	GG → YBRUN	−0.015	−0.352	0.725	Not supported
H2	GG → GN	−0.212	−3.527	<0.001	Supported
H3	GG → Gİ	0.138	2.401	<0.05	Supported
H4	Gİ → YBRUN	0.715	14.196	<0.001	Supported
H5	GN → YBRUN	−0.225	−4.932	<0.001	Supported

Note. GG: perceived future food insecurity; GN: food neophobia; Gİ: food interest; YBRUN: intention to incorporate edible insects into recipes. R^2^: Gİ = 0.019; GN = 0.045; YBRUN = 0.568. Fit: χ^2^/df = 3.194; AGFI = 0.838; CFI = 0.926; RMSEA = 0.074; GFI = 0.870; SRMR = 0.080; TLI = 0.916. Authors’ own Elaboration.

**Table 5:** Bootstrap mediation results from separate single-mediator models.

Hypothesis	Relation	Indirect Effect	*p*	95% CI	Direct Effect	Type of Mediation
H6	GG → GN → YBRUN	0.079	0.040	[0.028, 0.120]	0.045 (*p* = 0.479)	Indirect-only mediation
H7	GG → Gİ → YBRUN	0.098	0.024	[0.036, 0.193]	0.025 (*p* = 0.686)	Indirect-only mediation

Note: Bias-corrected bootstrap (1000 resamples, 95% CI). GG: perceived future food insecurity; GN: food neophobia; Gİ: food interest; YBRUN: intention to incorporate edible insects into recipes. Both relationships are purely indirect mediations according to the typology of Zhao et al. [26]. The direct effect in Table 4 (β = −0.015) was obtained from the full structural model including all four variables; the direct effects in Table 5 (0.045 and 0.025) were obtained from single-mediator submodels in which each mediator was modeled separately. The difference in coefficients stems from this model difference; all three values are non-significant and are standardized coefficients. Authors’ own Elaboration.

## Data Availability

The data supporting the findings of this study are not publicly available due to privacy and ethical restrictions but are available from the corresponding author upon reasonable request.

## Abbreviations

The following abbreviations are used in this manuscript:

AGFI	Adjusted Goodness-of-Fit Index
AIC	Akaike Information Criterion
AMOS	Analysis of Moment Structures
AVE	Average Variance Extracted
CFA	Confirmatory Factor Analysis
CFI	Comparative Fit Index
CI	Confidence Interval
CMB	Common Method Bias
CR	Composite Reliability
df	Degrees of Freedom
GG	Perceived Future Food Insecurity
GN	Food Neophobia
GFI	Goodness-of-Fit Index
HTMT	Heterotrait–Monotrait Ratio
Gİ	Food Interest
PMT	Protection Motivation Theory
RMSEA	Root Mean Square Error of Approximation
SEM	Structural Equation Modeling
SRMR	Standardized Root Mean Square Residual
TLI	Tucker–Lewis Index
VIF	Variance Inflation Factor
YB (YBRUN)	Intention to Incorporate Edible Insects into Recipes
